# Asthma incidence can be influenced by climate change in Italy: findings from the GEIRD study—a climatological and epidemiological assessment

**DOI:** 10.1038/s41598-023-46423-2

**Published:** 2023-11-03

**Authors:** S. Bonomo, P. Marchetti, S. Fasola, R. Vesentini, A. Marcon, G. Ferrante, L. Antonicelli, S. Battaglia, R. Bono, G. Squillacioti, N. Murgia, P. Pirina, S. Villani, S. La Grutta, G. Verlato, G. Viegi

**Affiliations:** 1 CNR Institute of Environmental Geology and Geo-Engineering (CNR-IGAG), Montelibretti, Rome, Italy; 2 Department of Diagnostics and Public Health, University of Verona, Verona, Italy; 3 CNR Institute of Translational Pharmacology (CNR-IFT), Palermo, Italy; 4 Department of Surgical Sciences, Dentistry, Gynecology and Pediatrics, University of Verona, Verona, Italy; 5 Allergy Unit, Ospedali Riuniti, Ancona, Italy; 6 Dipartimento PROMISE, University of Palermo, Palermo, Italy; 7 Department of Public Health and Pediatrics, University of Turin, Turin, Italy; 8 Department of Environmental and Prevention Sciences, University of Ferrara, Ferrara, Italy; 9 Respiratory Unit, Sassari University, Sassari, Italy; 10 Department of Public Health, Experimental and Forensic Medicine, University of Pavia, Pavia, Italy; 11 CNR Institute of Clinical Physiology (CNR-IFC), Pisa, Italy

**Keywords:** Risk factors, Environmental impact

## Abstract

An association between climatic conditions and asthma incidence has been widely assumed. However, it is unclear whether climatic variations have a fingerprint on asthma dynamics over long time intervals. The aim of this study is to detect a possible correlation of the Summer North Atlantic Oscillation (S-NAO) index and the self-calibrated palmer drought severity index (scPDSI) with asthma incidence over the period from 1957 to 2006 in Italy. To this aim, an analysis of non-stationary and non-linear signals was performed on the time series of the Italian databases on respiratory health (ISAYA and GEIRD) including 36,255 individuals overall, S-NAO, and scPDSI indices to search for characteristic periodicities. The ISAYA (Italian Study on Asthma in Young Adults) and GEIRD (Gene Environment Interactions in Respiratory Diseases) studies collected information on respiratory health in general population samples, born between 1925 and 1989 and aged 20–84 years at the time of the interview, from 13 Italian centres. We found that annual asthma total incidence shared the same periodicity throughout the 1957–2006 time interval. Asthma incidence turned out to be correlated with the dynamics of the scPDSI, modulated by the S-NAO, sharing the same averaged 6 year-periodicity. Since climate patterns appear to influence asthma incidence, future studies aimed at elucidating the complex relationships between climate and asthma incidence are warranted.

## Introduction

### Asthma epidemiology

Asthma is one of the most frequent chronic respiratory diseases worldwide, affecting over 330 million people of all ethnic groups throughout all ages^1,2^. Two recent publications have reported data from the Global Asthma Report 2022 issued by the Global Asthma Network (www.globalasthmanetwork.org); according to García-Marcos et al.^3^, the prevalence of ever asthma was 10.5 and 7.6% in adolescents (13–14 years) and children (6–7 years), respectively, whilst, according to Mortimer et al.^4^, it was 4.4% (range 0.9–29.0% in different countries) in adults (38+7.5 years). Notably, an increasing trend of asthma prevalence has recently been reported in Italy both for the general population^5^ and for children^6^.

Using information regarding subjects randomly sampled from the general Italian population between 1991 and 2010 in the three population-based multicentre studies (ECRHS, ISAYA, and GEIRD), Pesce et al.^7^ reported that the average yearly rate of asthma incidence was 2.6/1000. The incidence rates were linearly increasing, with a percentage increase of + 3.9%, from 1940 up to the year 1995, when the rates begun to level off. The stabilization of asthma incidence was mainly due to a decrease in the rates of atopic asthma after 1995, while non-atopic asthma continued to increase. Similarly, Maio et al.^8^, in a longitudinal study of a general population sample living in Central Italy, carried out from 1991–1993 to 2009–2011, found an 1.8/1000 yearly incidence of asthma.

Asthma is thought to be determined by a combination of genetic and environmental factors^9^. The latter can contribute to develop and/or exacerbate the disease and are largely associated with low air quality conditions, both indoor (e.g. presence of allergens or second-hand smoke) and outdoor (e.g. allergens and air pollution^10,11^), high tropospheric ozone levels, and others^12–14^.

Under specific climatic conditions, such as droughts accompanied by dusty conditions and wildfires producing smoke and dust, asthma can get worse^15^. Asthma and other chronic respiratory diseases have been also related to exposure from Saharan dust^12^. Short-term studies focusing on the analysis of temperature variations led to a wide consensus on considering extremely high temperatures as a risk factor for respiratory-related mortality in warmer regions^16,17^. However, assessments on the relationship between long-term changes in the persistence and intensity of temperature and precipitation-related extremes and asthma death rates are scarce^18^. Further, scanty evidence exists on the impact of temperature and precipitation variations on asthma mortality burden on climatological time scales^19^. While some published studies have assessed the existence of a link between climatic fluctuations, for example in terms of changes in sea surface temperatures or sea level pressure, and the respiratory system^17,19,20^, there are few studies linking the variations of the indices quantifying internal modes of climate variability with the time variations of respiratory diseases (and of asthma specifically).

### Summer Mediterranean climate

The climate in the Mediterranean region is mainly characterized by mild, wet winters and hot, dry summers. However, the geomorphological characteristics with its many sharp orographic features, often close to the coastlines, and the presence of distinct basins and gulfs, islands, and peninsulas surrounding the Mediterranean Sea basin, as well as the influence of the middle latitude and tropical atmospheric circulation patterns translate into a distinctively complex climate. Giorgi^21^ found this region to be particularly responsive to projected climate change and identified it as a climate hot spot. In fact, the Coupled Model Intercomparison Project phase 3^22,23^ and phase 5 future projections for this region^24–26^ indicate very strong warming and reductions in precipitation during the summer season. Temperatures in the Mediterranean region are characterized by high spatial complexity and a pronounced seasonal cycle, and they are influenced by large-scale atmospheric circulation, land–sea interactions, and local processes^27,28^.

In the past 6 decades, an overall warming tendency has been reported^29,30^. The recent upward tendency is particularly pronounced in summer^30,31^; yet, on a centennial timescale, trends are highly significant in all seasons^32^. The increasing temperature trend of the twentieth century has been connected with a reorganization of atmospheric regimes^33,34^ attributed to a combination of greenhouse gas forcing and internal/natural variability^33,35^.

Several studies^36–38^ have evidenced a linkage between the long-term decrease in Mediterranean precipitation during the period mid-1970s to early-1990s, and the decadal variations of the North Atlantic Oscillation (NAO)^39–41^. The summer expression of the NAO (S-NAO)^42–44^ in its positive phase yields a stronger meridional sea level pressure gradient over the North Atlantic, an enhanced anticyclonic southern lobe with dry conditions over northwest Europe, and rather wet conditions over the central Mediterranean. On interannual to multidecadal timescales, SNAO variability can be linked to variations in North Atlantic sea surface temperature (SST). Observations and models indicate an association between the Atlantic Multidecadal Oscillation (AMO) and the SNAO for periods greater than 10 years such that a cold (warm) phase of the AMO corresponds a positive (negative) phase of the SNAO^42–44^. However, the current literature has not yet reached a full consensus on the impacts of the SNAO. This stems largely from the pronounced interannual to multidecadal variability of the observed SNAO^44^.

### Aim

By hypothesizing that annual asthma incidence in Italy might be in part influenced by the occurrence of dry and wet periods, our study aimed at investigating a possible link between climate variations in summertime extratropical North Atlantic pressure and relative soil dryness, respectively expressed by the S-NAO index and the self-calibrated palmer drought severity index (scPDSI), with annual asthma incidence in Italy over a relatively long time period (1957–2006), as estimated from the Gene-Environment Interactions in Respiratory Diseases (GEIRD) Project data^45^.

## Results

### Asthma incidence

Overall, 4004 subjects reported lifetime asthma (1820/19221 from ISAYA and 2184/17034 from GEIRD). Since 635 of them did not report the age at asthma onset (241/1820 from ISAYA and 394/2184 respectively), these 635 records were left out from the analyses. Moreover, to prevent underestimation of the asthma incidence rates, we also left out, with the same proportions, a random subset of subjects who did not report lifetime asthma: 2304/17401 subjects from ISAYA and 2679/14850 from GEIRD. Therefore, the final study population comprised 30,637 subjects, 16,676 from ISAYA and 13,961 from GEIRD.

Mean (SD) age of the participants was 34.1 (7.4) in the ISAYA study and 41.6 (12.8) years in the GEIRD study (*p* < 0.001, Kruskal-Wallis test). A total of 8349/16666 subjects (50.1%) were males in the ISAYA study (10 subjects did not report their gender), while 6746/13955 (48.3%) subjects were males in the GEIRD study (*p* = 0.002, Chi-squared test). Characteristics of asthma cases are reported in supplementary material (Table S1).

**Figure 1:**
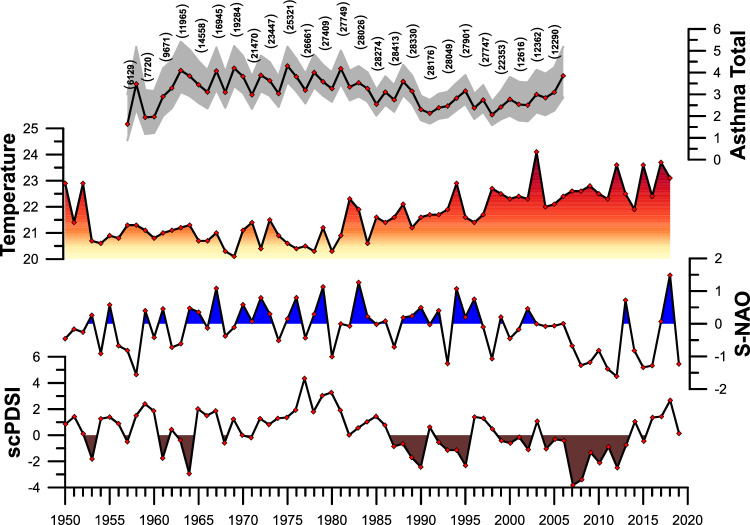
Comparison in time domain between the annual mean data of the self-calibrated palmer drought severity index (scPDSI), Summer North Atlantic Oscillation index (S-NAO), summer mean data of the Italy land Temperature, and annual asthma total incidence. Standard deviation (grey area), and case number (in brackets) were reported.

Asthma incidence variations for total participants are reported in Fig. 1 (values stratified by gender are reported in supplementary material). Incidence fluctuations were more pronounced till 1990, and an increasing trend was apparent in the third millennium.

### Climatic indices

The time-series of the average self-calibrated Palmer Draught Severity index (scPDSI) for Italy, the Summer North Atlantic Oscillation index (S-NAO), and the average summer temperature (ST) for Italy, from 1950 to 2020, are reported in Fig. 1. From 1950 to 1965 the scPDSI showed alternated positive (wet) and negative (dry) values, followed by a stable positive period from 1965 to 1986. Upwards, negative values occurred up to 2015, intermixed with short positive events, and again stable positive values from 2015 to 2020. The S-NAO is characterized by alternated positive (wet) and negative (dry) values from 1950 to 2005, followed by an 8-year interval characterized by persistent negative values up to 2013. Upwards, up to 2020 alternated positive and negative values occurred again. The ST time-series exhibits stable values (mean 20.8 °C) from 1953 to 1980, whilst, upwards, a positive trend can be observed up to 2020.

### Signal analysis

The CEEMD analysis revealed five intrinsic mode functions (IMFs), plus the trends (IMFs 6) (see supplementary material), for all analysed signals shown in Fig. 1. A first visual analysis of all IMFs shows that IMF2 and 3 for the asthma signals (total, male, female), the scPDSI, the S-NAO, and the TS recorded potentially significant periodicities. Instead, all IMF1 and 4 to 6 recorded predominantly noise or trends. For these reasons, the aforementioned IMFs were excluded from subsequent analyses. To obtain analytical results of periodicities recorded in the analysed signals, we applied REDFIT and Wavelet transform on a total of 12 IMFs and the spectra. Periodicities above 95% CI are reported in Table 1.

**Table 1:** Periodicity above 95% CI extracted from the IMF2 and IMF3, of the self-calibrated palmer drought severity index (scPDSI), Summer North Atlantic Oscillation index (S-NAO), summer mean data of the Italy land temperature (ST), and asthma incidence (total, male, female).

	IMF2	IMF3
scPDSI	6.3 yr (~1960 to ~2010)	21 yr
	9.4 yr (1950 to ~1980)	
ST	6 yr (1950 to ~1960/~1980 to ~1995)	–
	7.3 yr (1950 to ~2010)	
	10.3 yr (1950 to ~2010)	
S-NAO	5.8 yr (~1957 to ~2000)	–
	12.3 yr (~1990 to 2020)	
Asthma total	6.3 yr (~1957 to ~2000)	13.6 yr
	8.1 yr (~1980 to ~2000)	
Asthma male	5.2 yr (1957 to ~1980)	–
	6.2 yr (1957 to ~2000)	
	7.5 yr (~1980 to ~2000)	
Asthma female	6.3 yr (~1970 to ~2000)	10.7–15.10 yr
	10.7 yr (~1990 to 2006)	

**Figure 2:**
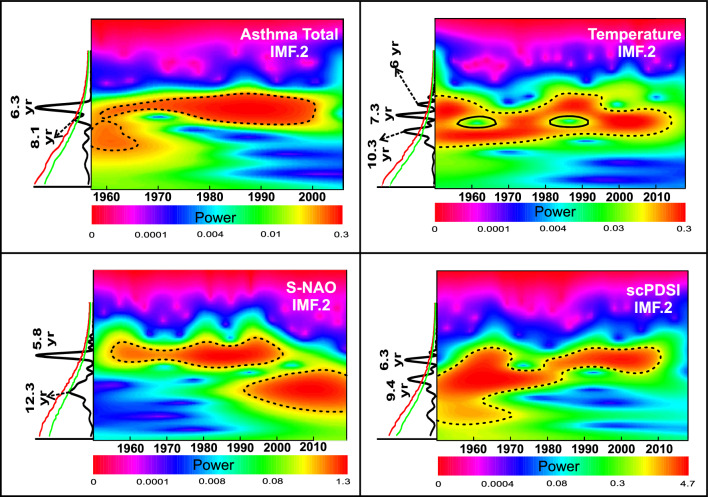
Signal analysis of the self-calibrated palmer drought severity index (scPDSI), Summer North Atlantic Oscillation index (S-NAO), summer mean data of the Italy land temperature, and annual asthma total incidence. In the 4 box IMFs2 Lomb-Scargle periodogram and continuous wavelet transform power spectrum were reported. The red and green line represent the 95% and 90% confident level respectively, black dash line represents the 95% confidence level. Significantly periodicity and relative values (expressed in years) were reported.

Indeed, only the IMF2 component in all analysed signals have main peaks in the ~6/~10 years periods range, all above the 90% and 95% IC (Fig. 2) (male and female asthma in supplementary material).

**Figure 3:**
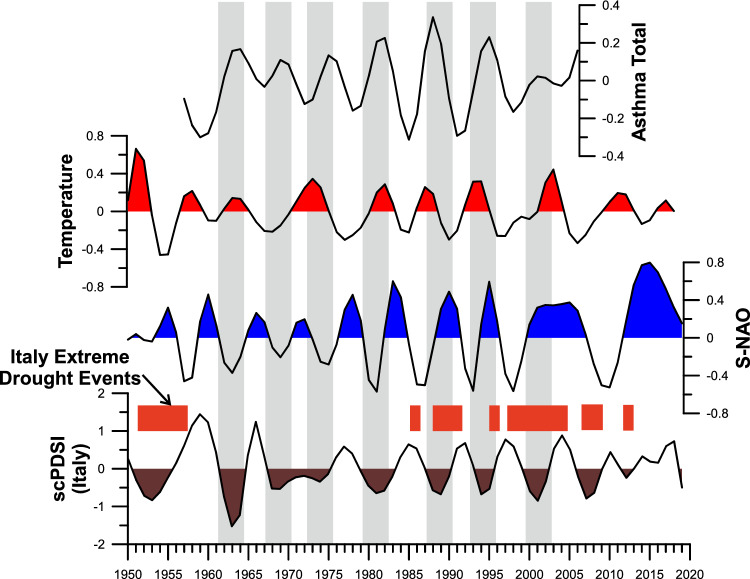
Comparison in time domain between the IMFs2 of the self-calibrated palmer drought severity index (scPDSI), Summer North Atlantic Oscillation index (S-NAO), summer mean data of the Italy land Temperature, and annual asthma total incidence. Italy extreme drought events (modified from Spinoni et al.^50^) were reported.

**Figure 4:**
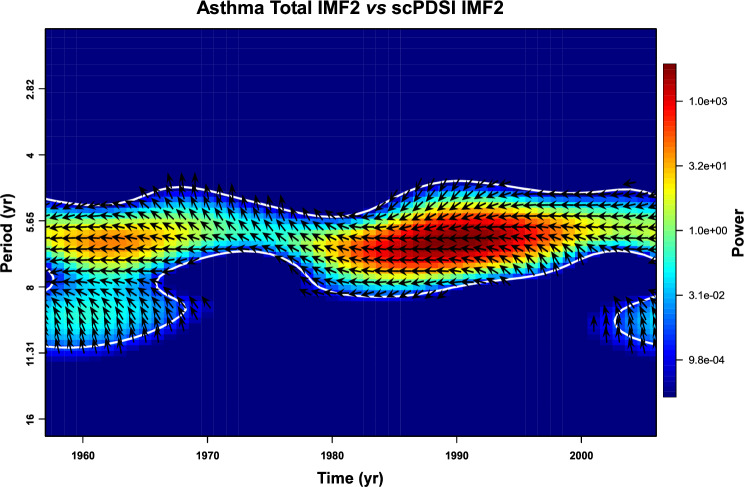
Cross-wavelet analysis between IMFs2 of the annual asthma total incidence and self-calibrated palmer drought severity index (scPDSI). The black arrows indicate the phase relationship between two IMF2 (right arrows = phase; left arrows = antiphase).

In Fig. 3, the IMF2s of asthma Total, scPDSI, S-NAO, and ST are depicted. The visual relationship of all IMF2 suggests constant synchronicity between negative (dry) scPDSI periods and positive asthma Total peaks. The visual relationship is confirmed by the correlation index (scPDSI vs asthma total r = − 0.69, *p*=4.8 × 10^–8^) and the cross wavelet analysis (Fig. 4). The comparison between all asthma data and S-NAO negative (dry) periods highlighted a comparable result with respect to scPDSI from 1957 to ~1985. After ~1985 and up to 2006, only the S-NAO positive (wet) periods were correlated to asthma Total peaks.

Finally, the ST IMF2 did not show a constant relationship with asthma Total IMF2s from 1957 to 1980. After 1980, a visual relationship between positive ST periods and asthma peaks occurred up to 2006. The IMF2 female (see supplementary material) was the only exception, showing in the 1998-2004 interval an evident and persistent period characterized by negative values versus a positive IMF2 ST interval.

## Discussion

The interactions among S-NAO, scPDSI, and the climatic conditions including the 1980–90 climatic shift, may have influenced and shaped the asthma incidence in Italy during the analysed time window.

This hypothesis is supported by the finding of a common mean periodicity of about 6 years among the analysed variables, which introduces some new elements in the research on asthma epidemiology, though some cyclical fluctuations have been already documented in the epidemiology of asthma^46^ and other diseases^47,48^.

This is the first study comparing historical Italian annual asthma incidence data with relevant climatic indices, which are known to modulate drought periods in this geographic area. Droughts conditions are among the main contributors to environmental factors leading to or exacerbating asthma^15,49^. The studied period covers a long time-span (50 years from 1957 to 2006), during which an increasing trend in surface air temperature has been observed and the historical records of precipitation, streamflow, and drought indices have pointed toward increased aridity since 1950 over many Europeans land areas^50–54^, including Italy.

Italian annual asthma incidence data have revealed a gradual increase in male and female. Moreover, all genders share the same increase/decrease sequences throughout this half a century, but a different long-term trend^55^. Since a long time-span of Italian annual asthma incidence has been described for the first time, our findings cannot be adequately compared to previous results. Nonetheless, results obtained for shorter time intervals are in agreement with our findings on increasing/decreasing sequences and trends^49,56,57^.

According to previous studies, asthma incidence variations might be attributed to different factors relative to prescription patterns^58^, medication misuse, underuse, overuse, air pollution exposures, as well as toxic effects^7,8,59^.

This study, taking advantage from the availability of the GEIRD project database, allowed us to explore, for the first time, the relationship between S-NAO, scPDSI and Italian annual asthma incidence over climatological timescales, therefore contributing to fill in an important knowledge gap. The correlation between increase/decrease sequences of asthma incidence and the S-NAO and scPDSI indices oscillation may indicate a phenomenon previously unrecognized in the link between climate variability and respiratory events. Indeed, we have observed a comparable evolution of the S-NAO and scPDSI and the fluctuations of annual asthma incidence in the studied period. Periods of maximum (minimum) in asthma incidence correspond to periods of negative (positive) values in the S-NAO and scPDSI indices, thus suggesting that the S-NAO, through its links to drought events, might be a risk factor for the Italian annual asthma incidence.

It is to point out the similarity of such results with those we obtained when we assessed the link between the Atlantic Multidecadal Oscillation and annual asthma mortality rates in the US^46^.

### Strengths and limitations

The present study took advantage of a robust signal analysis methodology applied to well known climatic indices and to a large national database on respiratory health, including 36,432 individuals from 12 centres located all over Italy, of whom 4004 reported asthma during their lifetime. The screening phase was performed by standardized questionnaires. As a limitation, asthma and year of first attack of asthma were self-reported; however, a good agreement was found between self-reported asthma and asthma assessed during the clinical visit in a subsample of 2194 individuals. Indeed, all cases of asthma ever, identified by questionnaire, were confirmed by the clinical visit, which also identified an additional number of 86 cases. Therefore, it can be argued that asthma ever was slightly underdiagnosed in the present study.

## Conclusion

Our findings suggest that patterns of climate variability may be an emerging risk factor for asthma. This is in agreement with the recent statement of the Global Asthma Network: “Environmental factors are much more likely than genetic factors to have caused the large increase in the number of people in the world with asthma, but we still do not know all the factors and how they interact with each other and with genes”^60^. Thus, it would be advisable to develop future correlative studies, including the aforementioned climatic indices, in order to elucidate the complex relationships between climatic factors and asthma.

At last, further collaboration between climatologists and epidemiologists is warranted in order to evaluate possible links regarding other chronic non-communicable diseases.

## Methods

### Study design and population

The current study includes 36452 subjects, aged 20–84, born between 1925 and 1989, who participated in three population-based multicentre surveys between 1998 and 2010. The three surveys, sharing the same design, were: the Italian Study on the Incidence of Asthma (ISIA) (n=3887)^61^, the Italian Study on Asthma in Young Adults (ISAYA, n=18,873)^62^, and the Genes Environmental Interaction in Respiratory Diseases (GEIRD) study (n=13,692) (S1 Text)^7,45^.

In all the aforementioned studies, a screening questionnaire enquiring about respiratory symptoms, past and present history of asthma, presence of allergic rhinitis and use of asthma drugs, was sent by post to 55,804 eligible individuals from the general population samples, with a 1:1 male-to-female ratio. Non-responders were contacted again, first by mail and then by phone, achieving a final response rate of 65.3%.

When pooling together the three databases, twenty subjects were excluded because date of birth (n = 5) and gender (n = 7) were discordant in two studies and 8 subjects filled-in the questionnaire twice. Hence, the sample size was reduced to 36,432 subjects.

A total of 3648 subjects from ISIA and ISAYA cohorts participated in a follow-up in the frame of the GEIRD study. For these subjects, we retained the most recent GEIRD questionnaire, unless the section related to asthma had been correctly completed only during the first survey. Hence, the study included 19,246 questionnaires from the ISAYA-ISIA surveys, collected in 1998–2000, and 17,186 were retained from the GEIRD survey, collected in 2005–2010, for a total of 36,432 subjects.

We also excluded 15 questionnaires with missing date of birth (10 from ISAYA and 5 from GEIRD), and 162 questionnaires for which the asthma section was left blank (15 from ISAYA and 147 from GEIRD). Hence, 36255 questionnaires were retained for the statistical analyses.

The presence of lifetime asthma and age at asthma onset were identified based on a positive answer to the following questions: “Have you ever had asthma?”; “How old were you when you had your first attack of asthma?”

Ethics approval was obtained in each centre participating in the GEIRD study from the appropriate ethics committee (Comitato Etico dell’Azienda Ospedaliero-Universitaria Ospedali Riuniti di Ancona; Comitato di Bioetica della Fondazione IRCCS Policlinico San Matteo di Pavia; Comitato Etico dell’Azienda Sanitaria Locale SA/2 di Salerno; Comitato di Bioetica dell’Azienda Sanitaria Locale di Sassari; Comitato Etico delle Aziende Sanitarie dell’Umbria di Perugia; Comitato Etico dell’Azienda Sanitaria Locale TO/2 di Torino; Comitato Etico per la Sperimentazione dell’Azienda Ospedaliera Istituti Ospitalieri di Verona). All the participants were informed about every aspect of the research, and informed consent was obtained. According to the Italian law, all methods were performed in accordance with the relevant guidelines and regulations, and respect of individual privacy concerning clinical data was guaranteed.

### Climatic indices

The monthly mean of S-NAO and scPDSI data (from 1950 to 2019) were downloaded from the National Oceanic and Atmospheric Administration (https://www.ncdc.noaa.gov/teleconnections/nao/) and the Climatic Research Unit (https://crudata.uea.ac.uk/cru/data/drought/), respectively. The scPDSI indicates the degree of drought severity (negative values = higher severity) and is based on climatic and environmental parameters. The scPDSI^63^ is a variant of the original PDSI of Palmer^64^, with the aim to make results from different climate regimes more comparable. As with the PDSI, the scPDSI is calculated from time series of precipitation and temperature, together with fixed parameters related to the soil/surface characteristics at each location.

In this study the scPDSI annual mean values were calculated by applying a 12-point running average. The S-NAO values were calculated by NAO (June, July, and August) mean values. Summer mean data of the land temperature throughout Italy (ST, 1950 to 2018) were downloaded from the Climatic Research Unit (https://crudata.uea.ac.uk/cru/data/temperature/).

### Statistical analyses

The characteristics of the study population were summarized through means and standard deviations (SD) for quantitative variables, and through absolute (No.) and percentage frequencies for categorical variables. A complete person-year dataset was created to compute asthma incidence rates by calendar year. Each subject entered the longitudinal database from start-year to end-year. The year of start was the date of birth for all the subjects. For subjects reporting lifetime asthma, the year of end was the year at asthma onset, calculated by adding the age at asthma onset to the start-year. For subjects who did not report lifetime asthma, the year of end was the year of questionnaire completion. For each subject and each year, we defined a binary variable “asthma”, which assumes value 1 for subjects reporting the asthma onset in that year, and 0 for all the other subjects.

Asthma incidence rates (‰) and 95% confidence intervals (IC) were, therefore, derived as proportions calculated on the asthma variable, stratified by year, age (attached to the long dataset as a time-dependent variable) and gender (time-invariant variable). Based on previous data^7^, the expected asthma incidence rate was about 2.6‰. The minimum sample size required to estimate such a proportion, through 95% CI with a desired width of at most 4‰ (± 2‰), was about 2500 individuals at risk for each year.

All the statistical analyses were carried out with R version 4.1.3 (R Foundation for Statistical Computing, Vienna, Austria).

### Signal analysis

In order to single out characteristic periodicities in the time-series, the analysis of non-stationary (frequency changes with time) and non-linear signals was performed through the application of the Ensemble Empirical Mode Decomposition algorithm (EEMD) by Wu and Huang^65^.

The EEMD is adaptive noise-assisted data analysis method that improves the ordinary Empirical Mode Decomposition (EMD) by Huang et al.^66^. Decomposition provides a powerful method to investigate the different processes behind a given time series data and separates short time-scale events from a general trend. This technique is based on the assumption that any complicated signal can be decomposed into a finite, often small, number of components defined as “Intrinsic Mode Functions” (IMFs)^66^.

Each IMF represents an embedded characteristic simple oscillation on a separated timescale. IMF components were analysed with “REDFIT”, Wavelet (WT), and Cross-Wavelet transform (XWT).

All data were detrended prior to analysis. All analyses were carried out with R (version 4.1.3) using the Rlibeemd^67^, dplR^68^, and Biwavelet^69^ packages.

### Supplementary Information

Supplementary Information.

## Data Availability

The asthma incidence datasets used and analysed in the current study are available from the corresponding author on reasonable request and with permission of the GEIRD Steering Committee.
