# Supplementary material for: Asthma incidence can be influenced by climate change in Italy: findings from the GEIRD study—a climatological and epidemiological assessment
Source: Sci Rep. 2023 Nov 3;13:19047. doi: 10.1038/s41598-023-46423-2 (PMC10624678; doi:10.1038/s41598-023-46423-2)
Supplement: Supplementary file 1 — Supplementary Information. [file 41598_2023_46423_MOESM1_ESM.pdf]

Supplementary information for

Asthma incidence can be influenced by climate change in Italy: findings from the GEIRD Study – A climatological and epidemiological assessment

Bonomo S.[1]*, Marchetti P.[2], Fasola S.[3], Vesentini R.[2], Marcon A.[2], Ferrante G.[4], Antonicelli L.[5], Battaglia S.[6], Bono R.[7], Squillacioti G.[7], Murgia N.[8], Pirina P.[9], Villani S.[10], La Grutta S.[3], Verlato G.[2], Viegi G.[11]

[1] CNR Institute of Environmental Geology and Geo-Engineering (CNR-IGAG), Montelibretti (Roma), Italy;

[2] Department of Diagnostics and Public Health, University of Verona, Italy;

[3] CNR Institute of Translational Pharmacology (CNR-IFT), Palermo, Italy;

[4] Department of Surgical Sciences, Dentistry, Gynecology and Pediatrics, University of Verona, Italy;

[5] Allergy Unit, Ospedali Riuniti, Ancona, Italy

[6] Dipartimento PROMISE, University of Palermo, Palermo, Italy

[7] Department of Public Health and Pediatrics, University of Turin, Torino, Italy

[8] Department of Environmental and Prevention Sciences, University of Ferrara, Ferrara, Italy

[9] Respiratory Unit, Sassari University, Sassari, Italy

[10] Department of Public Health, Experimental and Forensic Medicine, University of Pavia, Pavia, Italy

[11] CNR Institute of Clinical Physiology (CNR-IFC), Pisa, Italy.

*Corresponding Author: Dr. Sergio Bonomo - email: sergio.bonomo@cnr.it

Key words: Asthma; Incidence; Drought; S-NAO; sc-PDSI; Periodicity

Variables	n	Subjects with lifetime asthma n (%)
**Gender**		
**Male**	15095	1710 (11.3)
**Female**	15526	1657 (10.7)
**Centre**		
**Ancona**	1536	207 (13.3)
**Ferrara**	1815	161 (8.9)
**Modena**	1862	164 (8.8)
**Perugia**	1331	186 (14.0)
**Pisa**	2070	243 (11.7)
**Pavia**	3920	340 (8.7)
**Salerno**	1458	214 (14.7)
**Siracusa**	1057	84 (8.0)
**Sassari**	3495	470 (13.5)
**Torino**	4605	447 (9.7)
**Udine**	1807	150 (8.3)
**Verona**	5681	706 (12.4)
**Age at onset asthma (years)**		
**Median (25pct-75pct)**	3369	10 (4-23)

Table S1 – Characteristics of subjects included in the analysis.

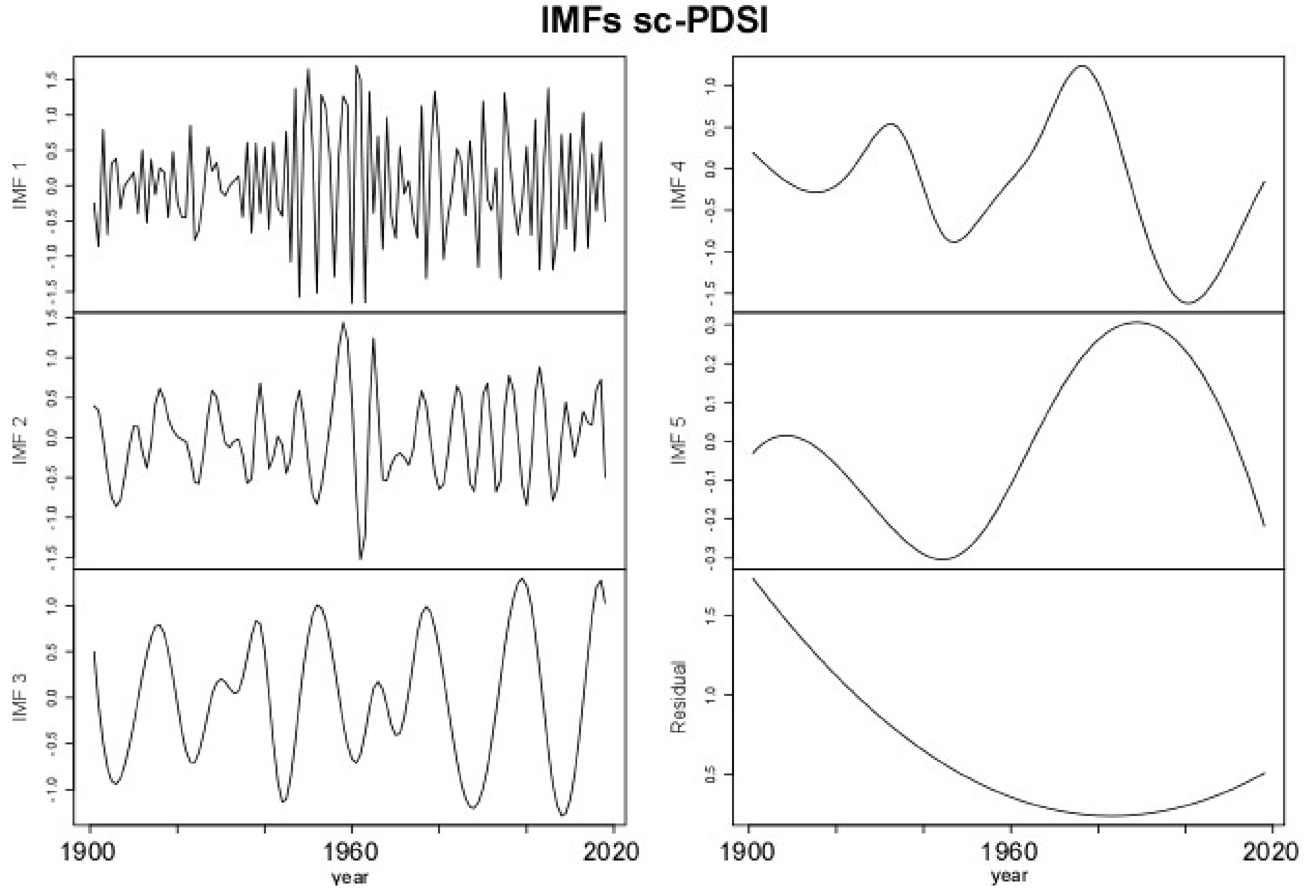

Figure S1. The self calibrated Palmer Drought Severity Index (sc-PDSI) Intrinsic Mode Functions" (IMF) diagrams. The IMF from 1 to 5 and Residual was reported.

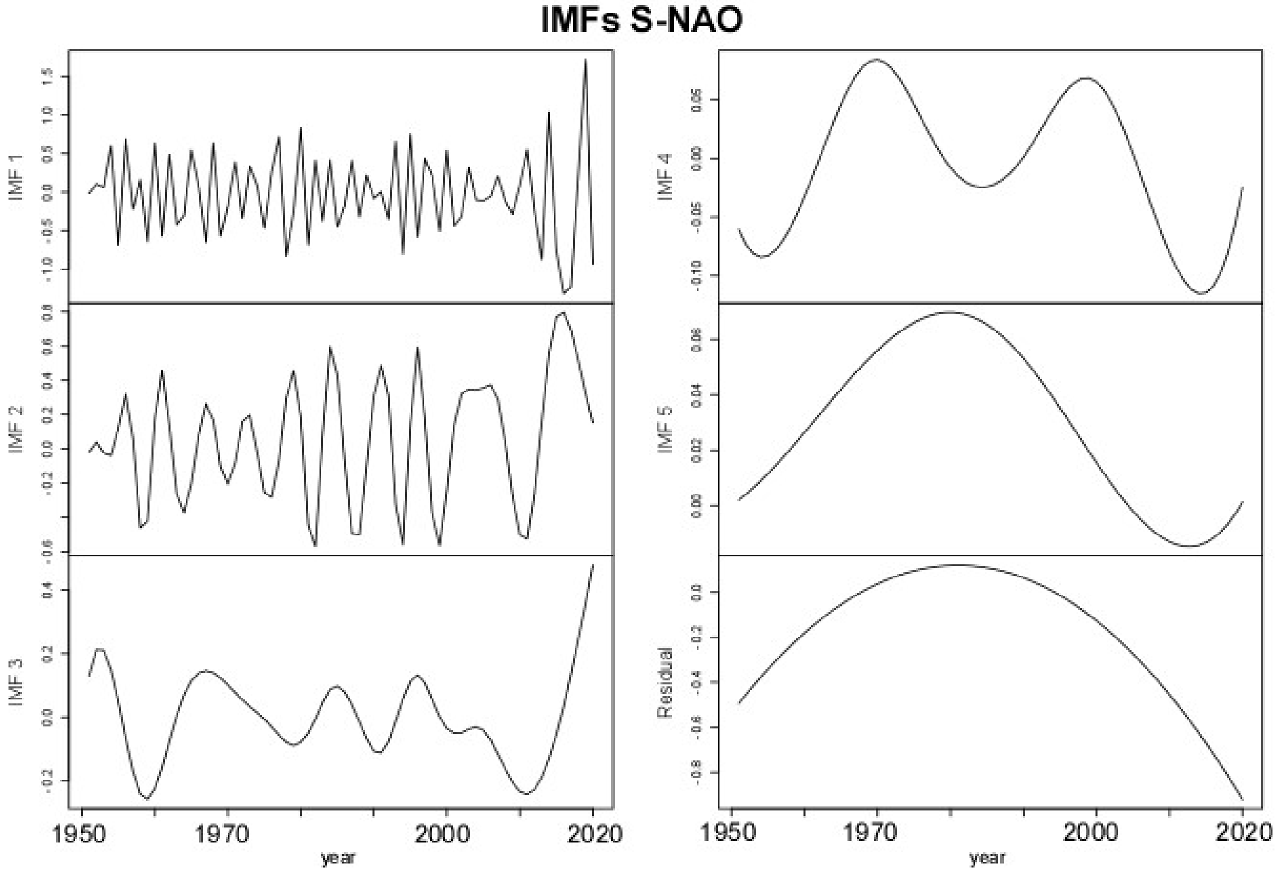

Figure S2. The Summer North Atlantic Oscillation Index (S-NAO) Intrinsic Mode Functions" (IMF) diagrams. The IMF from 1 to 5 and Residual was reported.

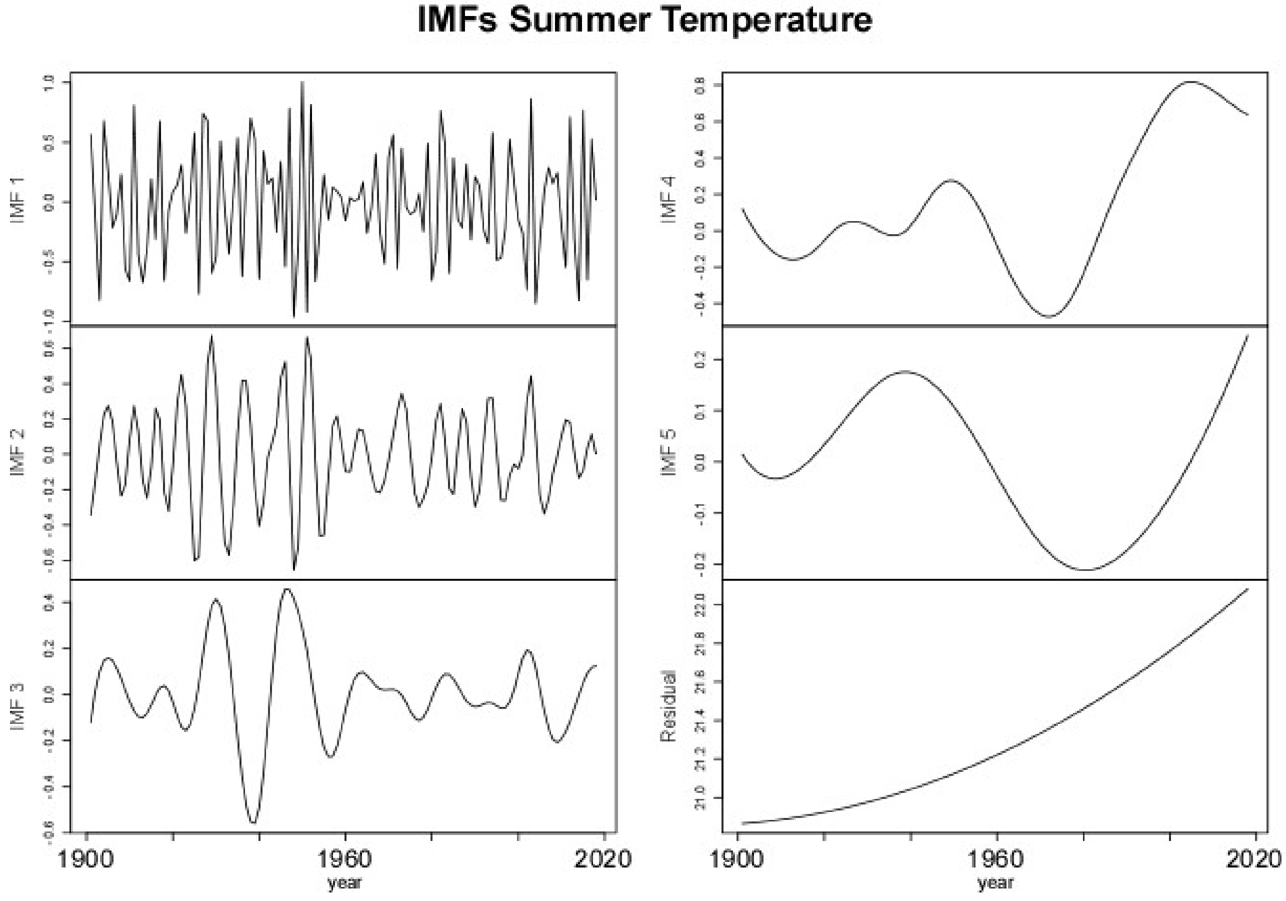

Figure S3. The Summer Temperature (ST) Intrinsic Mode Functions" (IMF) diagrams. The IMF from 1 to 5 and Residual was reported.

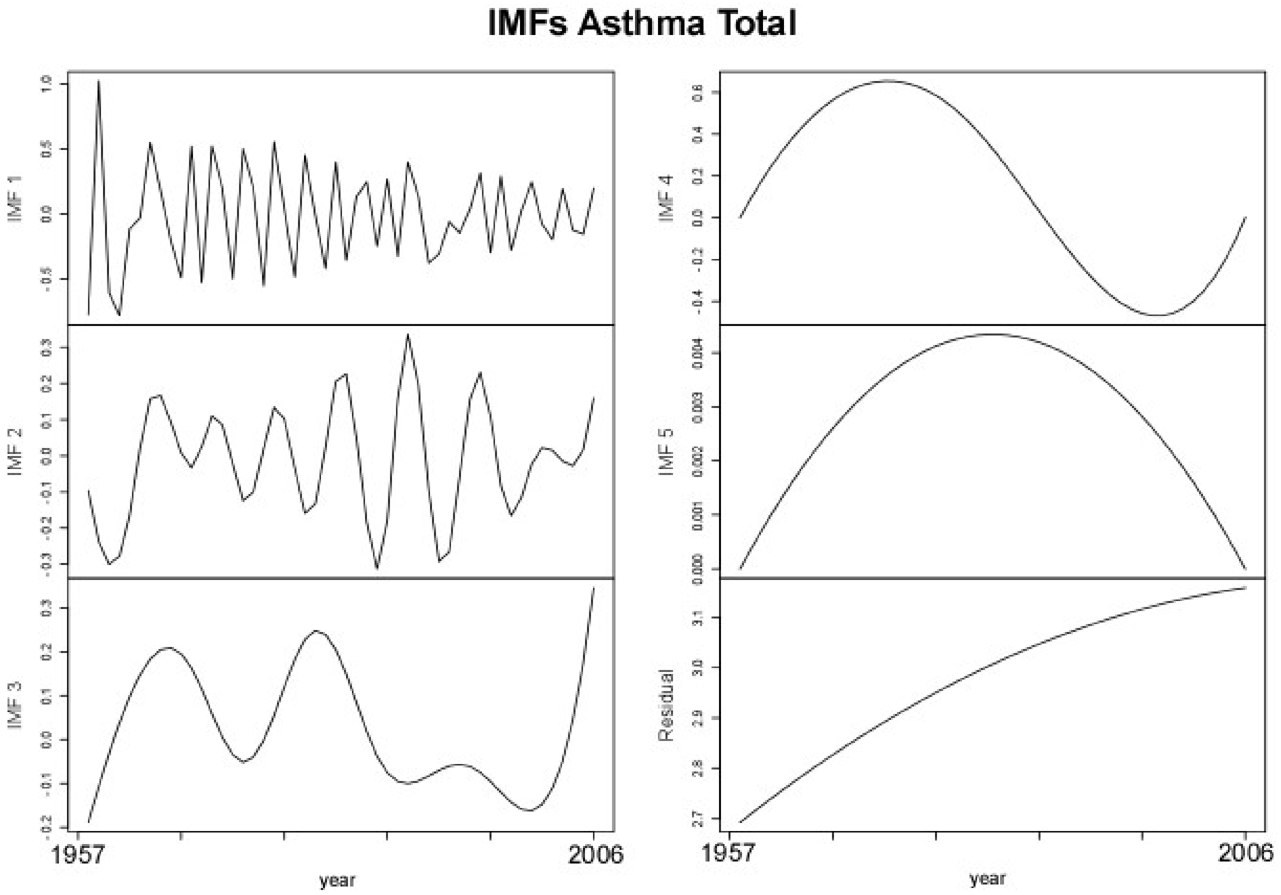

Figure S4. The Asthma Total Intrinsic Mode Functions" (IMF) diagrams. The IMF from 1 to 5 and Residual was reported.

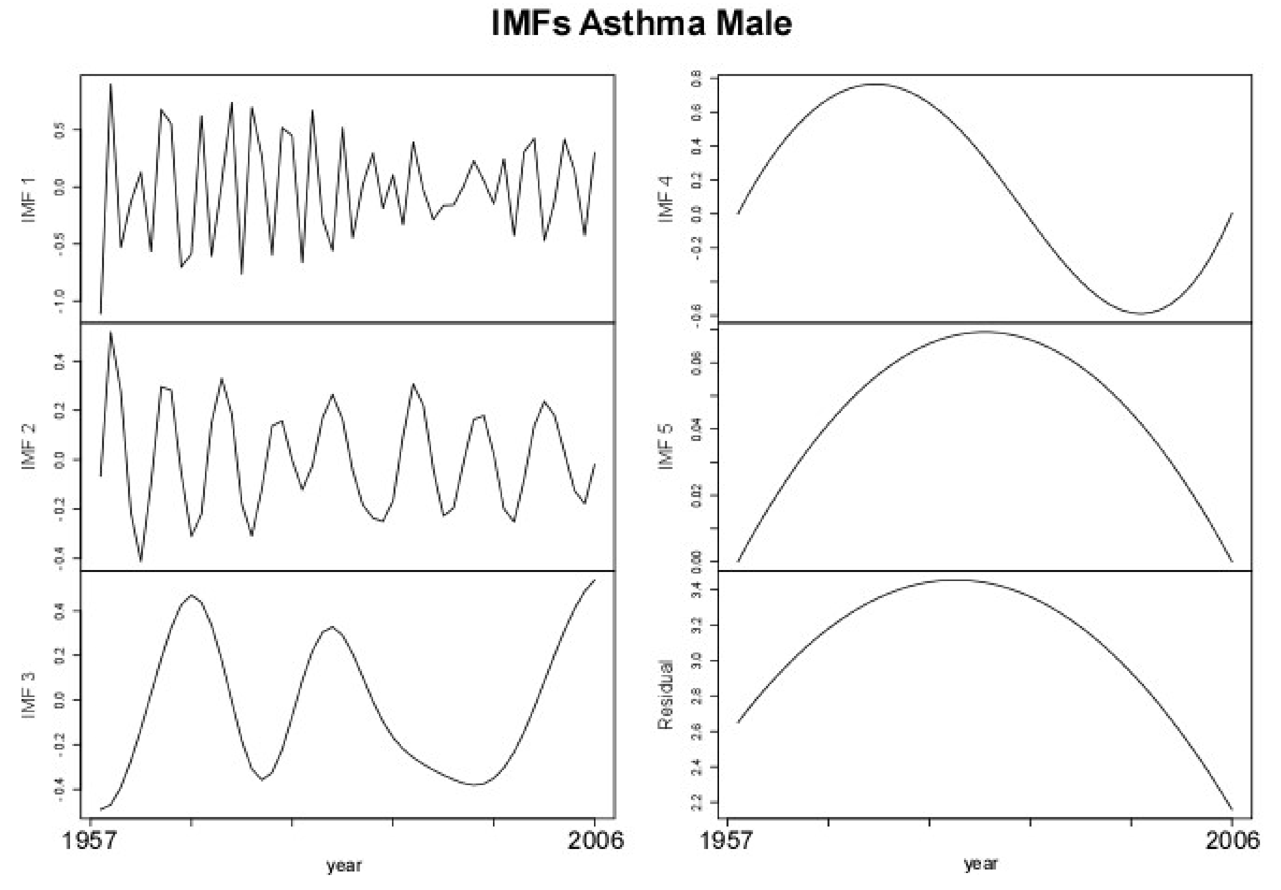

Figure S5. The Asthma Male Intrinsic Mode Functions" (IMF) diagrams. The IMF from 1 to 5 and Residual was reported.

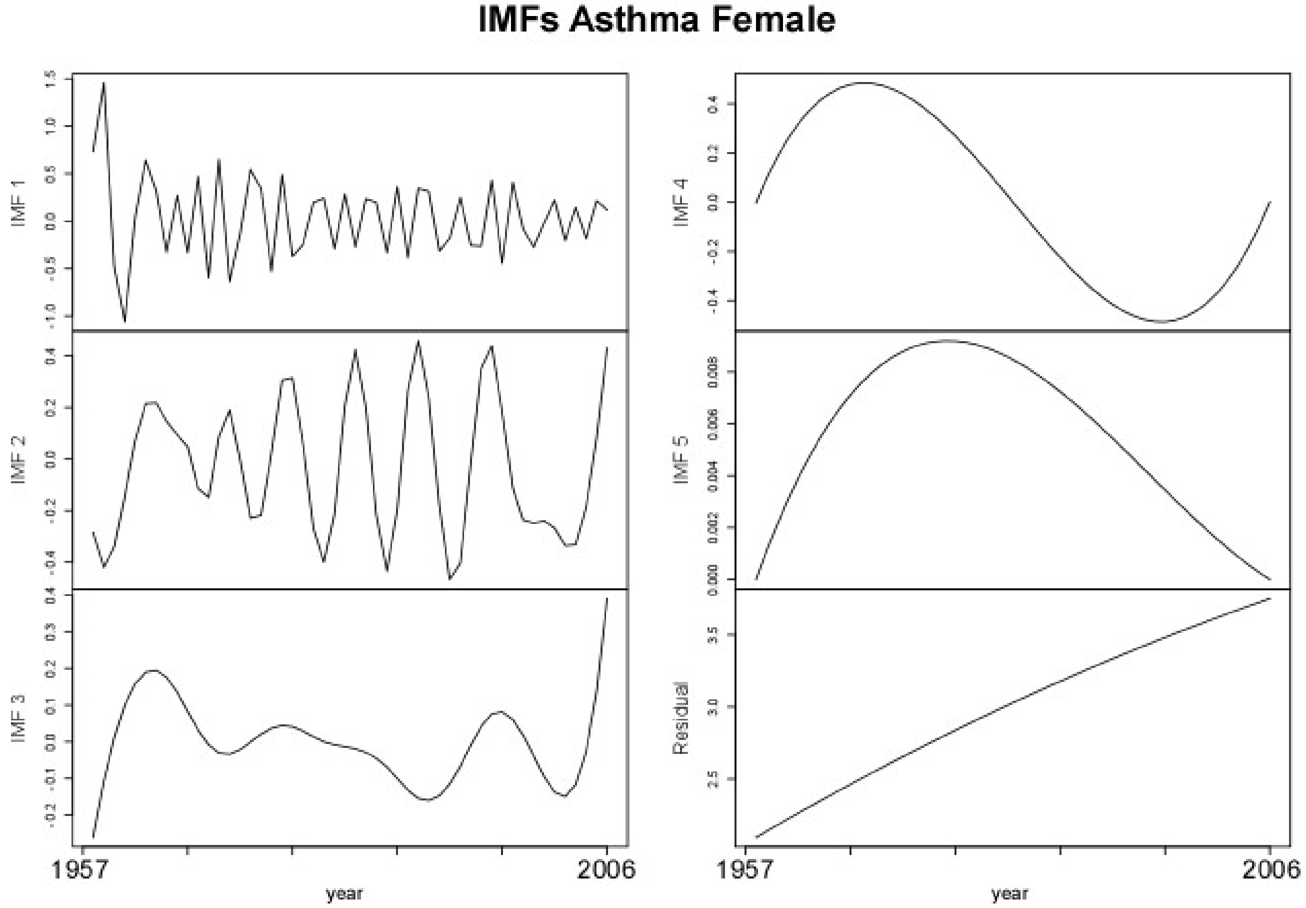

Figure S6. The Asthma Female Intrinsic Mode Functions" (IMF) diagrams. The IMF from 1 to 5 and Residual was reported.

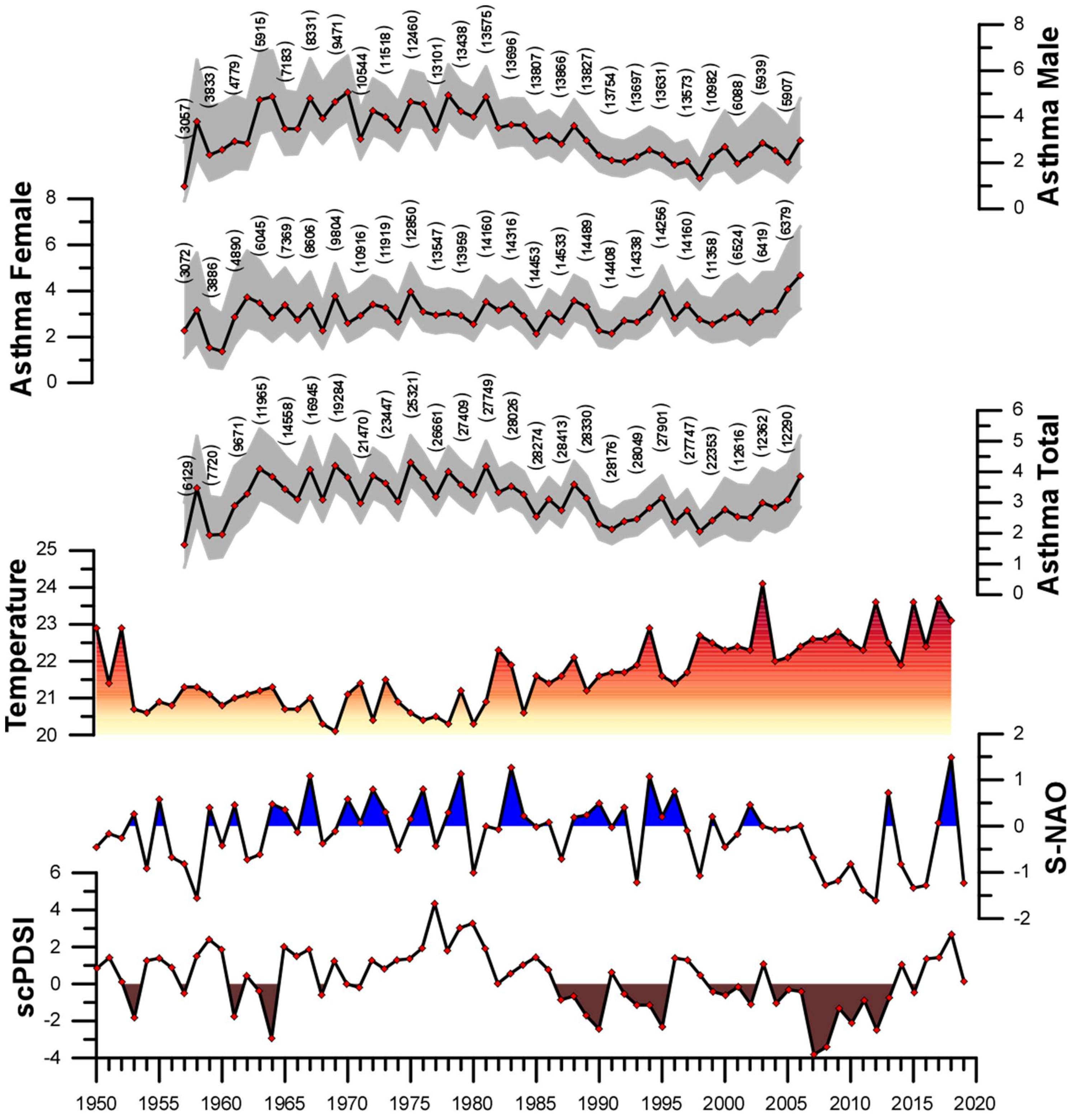

Figure S7. Comparison in time domain between the annual mean data of the self-calibrated Palmer Drought Severity index (scPDSI), Summer North Atlantic Oscillation index (S-NAO), summer mean data of the Italy land Temperature, and annual asthma total, female, and male incidence. Standard deviation (grey area), and case number (in brackets) were reported.

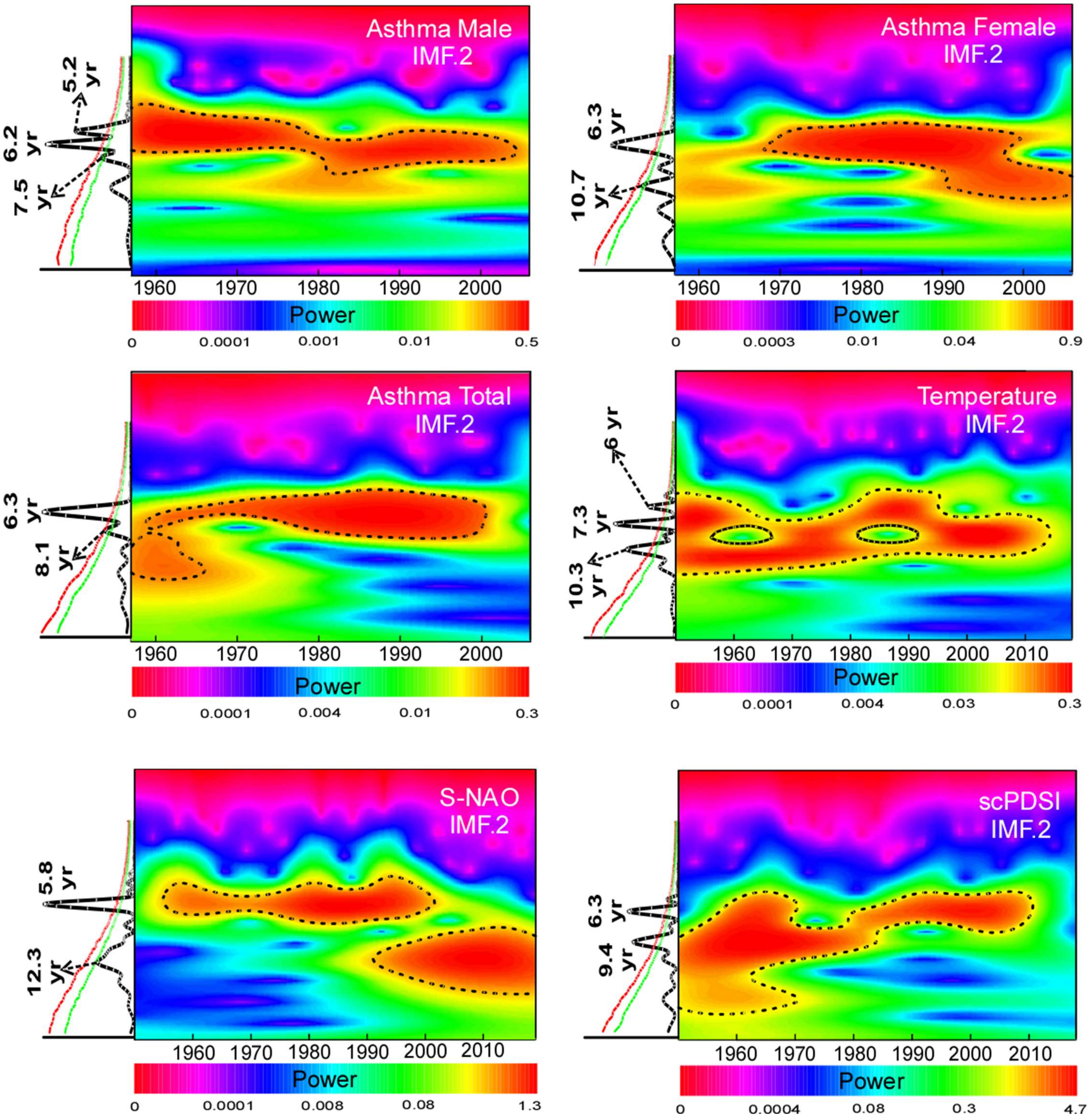

Figure S8. Signal analysis of the self-calibrated Palmer Drought Severity index (scPDSI), Summer North Atlantic Oscillation index (S-NAO), summer mean data of the Italy land Temperature, and annual asthma total, female, and male incidence. In the 6 box IMFs2 Lomb-Scargle periodogram and Continuous Wavelet transform power spectrum were reported. The red and green line represent the 95% and 90% Confident Level respectively, black dash line represent the 95% Confidence Level. Significantly periodicity and relative values (expressed in years) were reported.

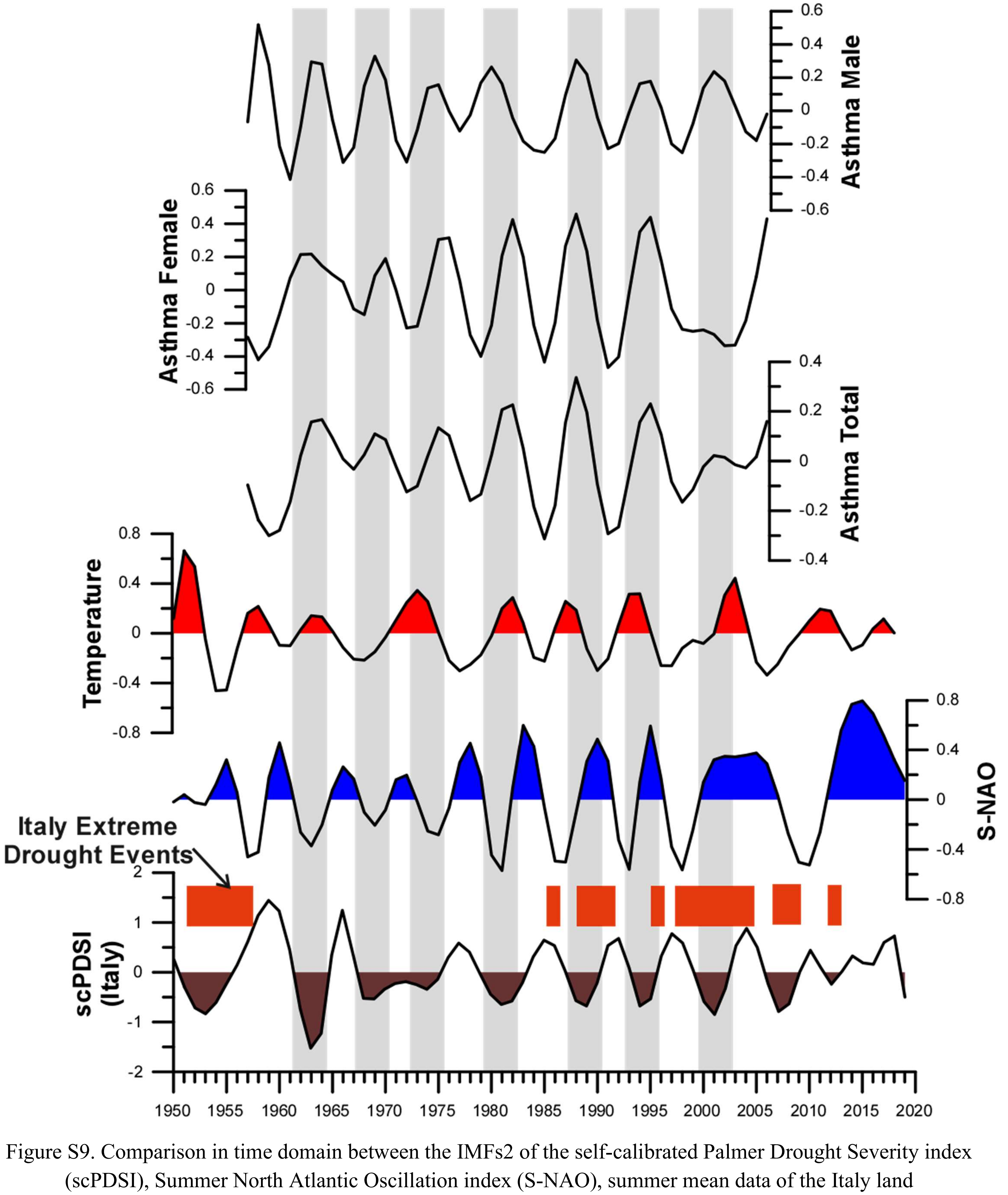

Figure S9. Comparison in time domain between the IMFs2 of the self-calibrated Palmer Drought Severity index (scPDSI), Summer North Atlantic Oscillation index (S-NAO), summer mean data of the Italy land Temperature, and annual asthma total, female, and male incidence. Italy extreme drought events (modified from Spinoni et al.[68]) were reported.
